# An Adaptive Deblurring Vehicle Detection Method for High-Speed Moving Drones: Resistance to Shake

**DOI:** 10.3390/e23101358

**Published:** 2021-10-18

**Authors:** Yan Liu, Jingwen Wang, Tiantian Qiu, Wenting Qi

**Affiliations:** School of Computer and Communication Engineering, Zhengzhou University of Light Industry, Zhengzhou 450000, China; 2015070@zzuli.edu.cn (J.W.); qiutiantian@zzuli.edu.cn (T.Q.); 331907040415@zzuli.edu.cn (W.Q.)

**Keywords:** unmanned aerial vehicle, vehicle detection, entropy, resistance to shake

## Abstract

Vehicle detection is an essential part of an intelligent traffic system, which is an important research field in drone application. Because unmanned aerial vehicles (UAVs) are rarely configured with stable camera platforms, aerial images are easily blurred. There is a challenge for detectors to accurately locate vehicles in blurred images in the target detection process. To improve the detection performance of blurred images, an end-to-end adaptive vehicle detection algorithm (DCNet) for drones is proposed in this article. First, the clarity evaluation module is used to determine adaptively whether the input image is a blurred image using improved information entropy. An improved GAN called Drone-GAN is proposed to enhance the vehicle features of blurred images. Extensive experiments were performed, the results of which show that the proposed method can detect both blurred and clear images well in poor environments (complex illumination and occlusion). The detector proposed achieves larger gains compared with SOTA detectors. The proposed method can enhance the vehicle feature details in blurred images effectively and improve the detection accuracy of blurred aerial images, which shows good performance with regard to resistance to shake.

## 1. Introduction

Unmanned aerial vehicles (UAVs) are increasingly used in plant protection [1], disaster relief [2], traffic monitoring [3], urban planning [4] and other fields [5,6,7,8,9] owing to their low cost, high flexibility, ease of operation and small size. The detection and classification of vehicles in aerial images is one of the important applications for drones, which is applied to the intelligent traffic system for traffic flow estimation and vehicle identification gradually, which is an application trend in the future.

Compared with the general vehicle detection tasks, the task for aerial images has some specific characteristics as follows. First, there are many small targets in aerial images, which are easily obscured by each other or the background. Second, drones generally have a camera lens with a large field of view (FOV), which may make the captured aerial images contain sparse and uneven target distributions. Finally, the images are liable to blurring since drones may shake when flying.

Researchers have proposed several algorithms aiming at these characteristics of aerial images. Lin et al. [10] designed a feature pyramid detection network, which implements the bottom-level features with more details and the top-level features with rich semantic information fusion. An image cascade network (ICN) was proposed by Azimi et al. [11] to make it possible to combine the image pyramid and feature pyramid models. In addition, a deformable network is used instead of a 1 × 1 convolution kernel in the feature pyramid to enhance the localization of small targets. LaLonde et al. [12] introduced a two-stage convolutional neural network (CNN) in their study of target detection in wide-area motion images. The first stage of the algorithm improves the region proposal network (RPN) in the Faster R-CNN to cover more potential objects; in the second stage, a high-resolution analysis is performed on the output of the first stage above a set threshold. For the problem of uneven distribution of targets in aerial images, Yang et al. [13] proposed a target detection algorithm for aggregated regions.

In this article, considering the inevitable blurring problem of aerial images caused by the high-speed movement of drones and vehicles, an end-to-end adaptive deblurring vehicle detector (DCNet) is proposed. The major contributions of this article are summarized as follows:

1. A dataset consisting of blurred and sharp image pairs is provided in this article.
2. A clarity evaluation (CE) module based on information entropy is introduced to discriminate whether an image is blurred or not.
3. An improved GAN called Drone-GAN is proposed to improve the detection accuracy of blurred aerial images.
4. The algorithm proposed can be applied to the target detection task with a high-speed camera platform.

## 2. Related Works

Vehicle detection based on aerial images [14,15,16] is extremely important for some fields such as intelligent traffic management. Current methods of vehicle detection for drones are divided into the traditional methods and the methods based on deep learning. In the traditional vehicle detection algorithms [17,18,19,20,21,22,23,24,25], the detection methods based on handcrafted feature extraction dominate. The method consists of three stages. Firstly, finding vehicle-like regions using methods such as sliding windows and superpixels. Secondly, features are manually extracted from these regions. Finally, the classifier is used for classification and recognition. The traditional detection algorithms need to manually obtain relevant target feature information so that they are accompanied by many limitations. First, the portability is poor because specific inspection tasks require different manually designed feature extraction methods. Second, the traditional methods mostly use sliding windows for traversal search, which has high complexity and a large amount of redundancy that affects the operation speed.

Significant progress has been made in aerial image vehicle detection based on deep learning [26,27,28,29,30,31,32,33]. Due to the characteristics of aerial images (low resolution, blurred image, small targets with little information and high optical noise), the identification of small targets is always a challenge in the aerial image target detection task. Considerable amount of research has been conducted to improve the accuracy of target detection. Rabbi et al. [34] proposed a three-part network structure to improve the resolution of aerial images. A multiscale pyramid for the training images was proposed by Bharat Singh et al. [35] through resizing the training images. A joint generative adversarial network was proposed by Moktari et al. [36] to improve the resolution of aerial images through a multiscale operation and enhance the feature recognition capabilities. A method was proposed by Mandal et al. [37] to enrich the feature maps of aerial images, which preserves the features of small targets by using ConvRes blocks in diverse scale layers. A method for detecting multiscale targets was proposed by Lin et al. [10], which uses feature pyramids to select the best detection results. The abovementioned methods based on pyramid algorithms are mainly focused on clearing the image.

UAVs rarely carry a stable imaging platform because they are easily affected by airflow disturbances, high-speed motion of captured objects and the flight motion of the aircraft, resulting in blurred images. The current methods are mainly focused on improving the resolution of the image and enriching the detailed information of the feature maps described by the target, while few works have addressed the problems brought by motion blurring of aerial images in the vehicle detection process. Therefore, an adaptive deblurring vehicle detection method for high-speed moving drones is proposed in this article to resist shake.

## 3. Method

**Figure 1:**
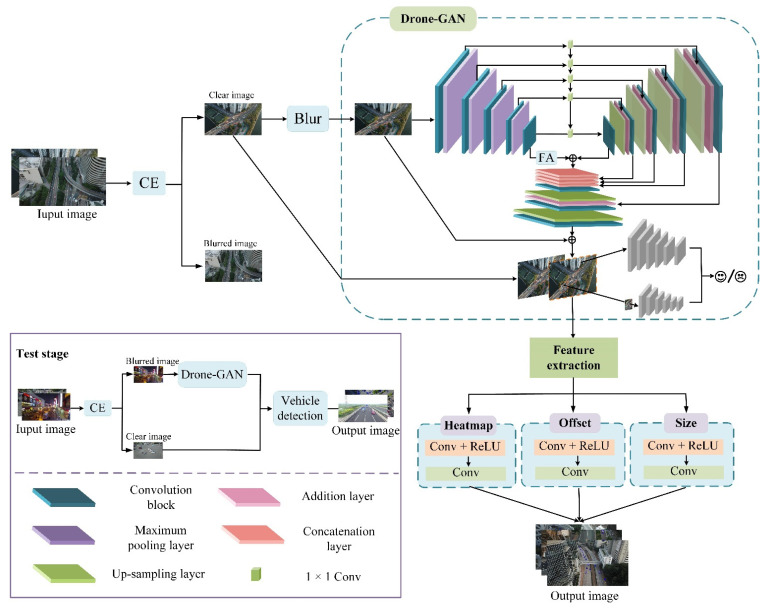
The DCNet architecture.

The proposed method as shown in Figure 1, which is called DCNet in this article, includes four modules: clarity evaluation (CE) module, blurring dataset construction module, Drone-GAN module and vehicle detection module. In the training phase, the inputs are divided into blurred and clear images through the clarity evaluation module, and then the clear images are divided into blurred and clear image pairs through the blurred dataset construction module. Then, Drone-GAN is introduced to enhance the vehicle features in the images. Finally, small vehicles are detected by the vehicle detection module. In the test phase, if the inputs are classified as blurred images by the evaluation module, the blurred images are enhanced by Drone-GAN for blurred vehicle features. All the inputs enter the vehicle detection module for the detection results to be obtained. The proposed algorithm is depicted in the following subsections in detail.

### 3.1. CE

In the proposed DCNet network, improved information entropy is used in the clarity evaluation module, in which the Sobel operator [38] is combined with information entropy [39] to detect the blur of the image. Then, the entropy value of the image is calculated. The improved information entropy calculation value can more accurately distinguish between clear and blurred images. Especially for slightly blurred images, the blur can be more accurately judged. When the image is clear, the clarity evaluation function of information entropy is directly used, the pixel gray value distribution interval is wide, the difference between the gray values is large and the entropy value is large. However, when the image is slightly blurred, the gray value of the image can still be distributed in a wide range and the difference is large. In that case, it is not possible to judge whether the image is blurred. Improved information entropy can transform the image into a binary edge detection image, which solves the problem of misjudgment of slightly blurred images due to the direct use of information entropy and increases the accuracy of judging clear and blurred images. The improved information entropy function is defined as Equation (1):

(1) $$D=-\overset{M}{\underset{x=1}{\sum }}\overset{N}{\underset{y=1}{\sum }}H\left(x,y\right)\log H\left(x,y\right)$$

where D is the calculation result of improved information entropy, the Sobel operator S is used to detect the edge of the image I; let the convolution result of S and I be H.

The larger the entropy value, the clearer the image (improved information entropy). We chose the maximum improved entropy value of the blurred image dataset as the threshold between the clear and blurred images. Table 1 shows the entropy values of clear and blurred images, and the threshold is 5.5.

### 3.2. Blurred Image Dataset

**Figure 2:**
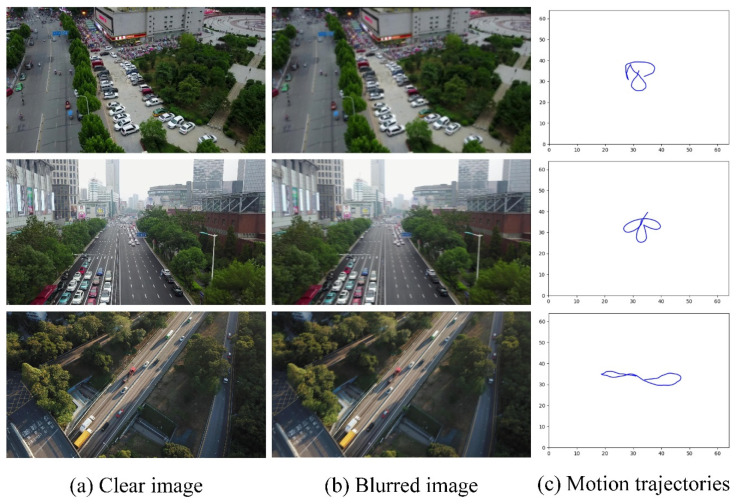
Examples of generated camera motion trajectories and the corresponding blurred images. Column (**a**) represents clear images, column (**b**) represents blurred images and column (**c**) is the motion trajectory of the camera.

A simulated realistic and complex blur kernel [40] is introduced to produce blurred image datasets. We adopted the idea of random trajectory generation described by Boracchi and Foi [41]. Then, the kernel is generated using the interpolation operation to the sub-pixel on the trajectory vector. Finally, we obtain a blurred image by convolving the kernel with the clear image. The blurred dataset is shown in Figure 2.

### 3.3. Drone-GAN

GAN [42] networks are usually composed of two modules, a generator and a discriminator, respectively.

In this article, Inception-ResNet-v2 [43] and the improved feature pyramid network constitute the generator. Feature reuse in the feature pyramid structure can greatly reduce the computation time and model size. Five final feature maps of different scales are used as the outputs of the generator, the features of which are up-sampled to the same as 1/4 of the input size and join into a tensor containing different levels of semantic information. Two additional up-sampling and convolution layers are added in the end network to recover the size of the original image and reduce the artifacts. Finally, a connection is skipped from the input to the output directly to focus on the residual.

**Figure 3:**
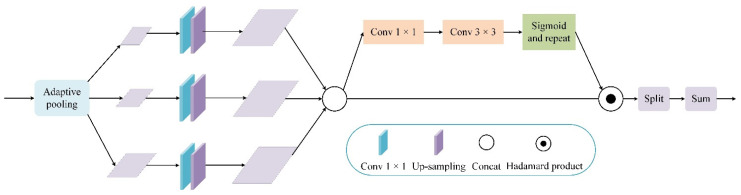
FA module.

In the top–down stage of the feature pyramid network (FPN) [10], the highest-level feature map causes information loss due to the reduction of feature channels and only single-scale deep semantic information. Therefore, we propose a feature augmentation (FA) module to improve this problem. In the feature augmentation module, multiple context features of different scales are first generated by using adaptive pooling on the input feature map. Then, 1 × 1 convolution is performed independently for each context feature to reduce the dimension of the feature channel to 256. Up-sampling is performed on the reduced dimensional features. Finally, the spatial weight map of each feature is generated after up-sampling using the weight aggregate of the contextual features as the output of the feature enhancement module, which has multiscale contextual information. The spatial context information provided by the feature augmentation module reduces information loss in the FPN and improves the performance of the resulting feature pyramid. The feature augmentation module is shown in Figure 3.

To take full advantage of the local and global features, a dual-scale discriminator is introduced. We adopted a relativistic discriminator [44] wrapped in least-squares loss [45] and used two columns to compute the global (image) and local (patch) scales [46] separately. Using the dual-scale discriminator can make the generated clear images more realistic. The loss function of the discriminator D is described by Equation (2):

(2) $$L_{D}=E_{x∼p_{data}\left(x\right)}\left[{\left(D\left(x\right)-E_{z∼p_{z}\left(z\right)}D\left(G\left(z\right)\right)-1\right)}^{2}\right]\;+E_{z∼p_{z}(z)}\left[{\left(D\left(G\left(z\right)\right)-E_{x∼p_{data}\left(x\right)}D\left(x\right)+1\right)}^{2}\right]$$

where G is the generator, D is the discriminator and z is the noise, $p_{data}\left(x\right)$ is the probability distribution that the real data x obeys, $p_{z}\left(z\right)$ is the probability distribution that z obeys; $E_{x∼p_{data}\left(x\right)}$ and $E_{z∼p_{z}(z)}$ are the expected values.

The loss function of the generator G is as follows:

(3) $$L_{G}=0.5*L_{m}+0.006*L_{x}+0.01*L_{adv}$$

where $L_{m}$ is the mean square error loss which helps correct color and texture distortion, $L_{x}$ is the content loss using the perceptual distance; $L_{adv}$ includes the global and local discriminator loss.

### 3.4. Vehicle Detection

The vehicle detector in this article is a single-stage anchor-free detector based on CenterNet [47], the anchor points of which are extracted from the heatmap in which each target corresponds to only one anchor point. Hence, there is no need in NMS [48] to filter the anchor points, which can speed up the detection process. Compared with the anchor-based method, the detection problem is solved by the predefined anchor points, which lead to the lack of robustness and detection accuracy for multiscale detection. In addition, the output resolution of the detector has a down-sampling factor of 4, which is relatively small compared to other target detection frameworks. Such a setting results in a larger output feature map resolution, helping small targets detection.

**Figure 4:**
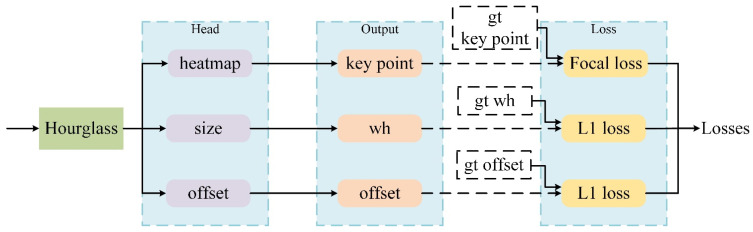
The framework of vehicle detection.

Vehicle detection part is composed of two parts: the feature extraction part and the detection head, as shown in Figure 4. The detection head consists of three parts: heatmap, offset and size. The size of the input image is 512 × 512. Hourglass [49] is used for feature extraction to obtain a feature map with a size of 128 × 128. After the output of the feature map, a 3 × 3 convolution is added in front of each output header, followed by a 1 × 1 convolution operation to obtain the desired output. The offset module presents the offset of the center point. The size module denotes the height and width of the target bounding box. The number of channels in the heatmap equals the number of detected target classes. The first 100 peak values of the heatmap are extracted by the network for use as the target center points, and then the threshold value is determined to filter the final target center points. The target is represented by its center point, and then some attributes of the target are returned at the center point.

The hm branch objects are represented by a Gaussian kernel, the pixel values of which are mapped to the [0,1] range through the sigmoid operation, so the Gaussian kernel is shown in Equation (4):

(4) $$Y_{xyc}=\exp\left(-\frac{{\left(x-{\tilde{P}}_{x}\right)}^{2}+{\left(y-{\tilde{P}}_{y}\right)}^{2}}{2\sigma_{p}^{2}}\right)$$

where $Y_{xyc}$ denotes the Gaussian kernel, $\left({\tilde{P}}_{x},{\tilde{P}}_{y}\right)$ is the kernel centerpoint and $\sigma_{p}$ is the standard deviation based on the object bounding box size.

The focal loss [50] is used to optimize the prediction of the hm branch heatmap from the output in the vehicle detection procedure. The loss function is described as Equation (5):

(5) $$L_{hm}=\frac{-1}{N}\underset{xyc}{\sum }\{\begin{array}{cc} {\left(1-{\tilde{Y}}_{xyc}\right)}^{\delta }\log\left({\tilde{Y}}_{xyc}\right) & Y_{xyc}=1\\ {\left(1-Y_{xyc}\right)}^{\rho }{\left({\tilde{Y}}_{xyc}\right)}^{\delta }\log\left(1-{\tilde{Y}}_{xyc}\right) & else \end{array}$$

where N is the number of key points in image I, the ground truth of the Gaussian kernel is represented by $Y_{xyc}$, ${\tilde{Y}}_{xyc}$ is the predicted value of the Gaussian kernel, δ and ρ represent hyperparameters of the focal loss. We assign δ and ρ the values of 2 and 4, respectively.

In order to reduce the dispersion error induced by the output step, the offset loss t value is used to represent the distance between the predicted target center point and the ground truth. The offset loss function can be expressed as follows:

(6) $$L_{off}=\frac{1}{N}\underset{P}{\sum }\left|{\hat{O}}_{\tilde{P}}-\left(\frac{P}{R}-\tilde{P}\right)\right|$$

where ${\hat{O}}_{\tilde{P}}$ is the predicted offset, $\tilde{p}$ and P are the predicted and ground truth values of the target center, respectively, and R is the stride of the predicted heatmaps.

Let $\left(x_{1}^{\left(k\right)},y_{1}^{\left(k\right)},x_{2}^{\left(k\right)},y_{2}^{\left(k\right)}\right)$ be the bounding box of object K, object *k* belongs to category $c_{k}$, $s_{k}=\left(x_{2}^{\left(k\right)}-x_{1}^{\left(k\right)},y_{2}^{\left(k\right)}-y_{1}^{\left(k\right)}\right)$ is the ground truth size of object K and ${\hat{S}}_{pk}$ denotes the positive prediction of the size of object K. Equation (7) represents the size loss function.

(7) $$L_{size}=\frac{1}{N}\overset{N}{\underset{k=1}{\sum }}\left|{\hat{S}}_{pk}-s_{k}\right|$$

Finally, different weights are applied to $L_{off}$ and $L_{size}$, and the total loss of vehicle detection can be expressed as Equation (8).

(8) $$L_{vd}=L_{hm}+\lambda_{off}L_{off}+\lambda_{size}L_{size}$$

In this article, we set $L_{off}$ = 1 and $L_{size}$ = 0.1.

### 3.5. Implementation

The method proposed in this article is based on Python programming and the PyTorch deep-learning framework. In order to run the algorithm, we built the deep-learning environment for PyTorch on the basis of the Windows 10 operating system, i.e., CUDA v10.0 + cuDNN v7.4.1.5 + PyTorch. All the experiments were performed in the Windows 10 operating system with 32 GB RAM and 3.6 GHz CPU, NVIDIA GeForce RTX 2080 Ti 11 GB GPU.

## 4. Experiments

### 4.1. Datasets and Evaluation Metrics

**Figure 5:**
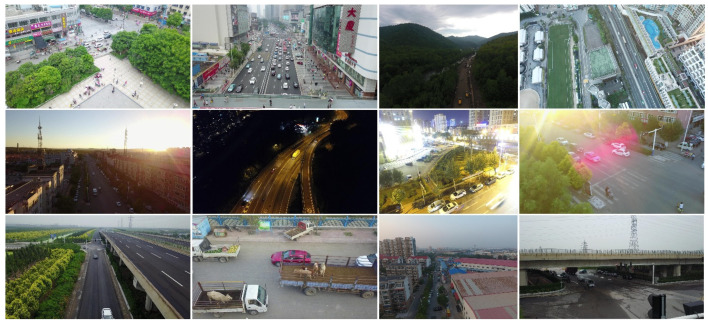
VisDrone dataset.

To validate our proposed method, the experiments were performed on the large-scale UAV target detection and tracking benchmark VisDrone 2019 DET dataset [51]. The VisDrone dataset consists of multiple road scene images with large object size variations, rich image variations and high interclass similarity. However, there is a small number of blurred images in VisDrone. Hence, the training dataset and the test dataset in this article were constructed by randomly selected images in VisDrone. There were 7019 and 1610 images in the training dataset and the test dataset, respectively. The test set had 998 clear images, 332 real blurred images and 280 simulated blurred images. A brief demonstration of the dataset is shown in Figure 5.

The mean average precision (mAP) is the target detection quality evaluation metric employed in this article, which is the average value of multiple categories of average precision (AP). The AP is the area under the P_R curve, which can be gained by precision and recall. The formulas are as follows:

(9) $$Precision=\frac{TP}{TP+FP}$$

(10) $$Recall=\frac{TP}{TP+FN}$$

where TP denotes a true-positive sample, TN represent a true-negative sample, FP present a false-positive sample, and FN depicts a false-negative sample. Precision indicates the number of recalled positive samples. On the other hand, the recall rate indicates the number of recalled true-positive samples.

### 4.2. Experimental Results

**Figure 6:**
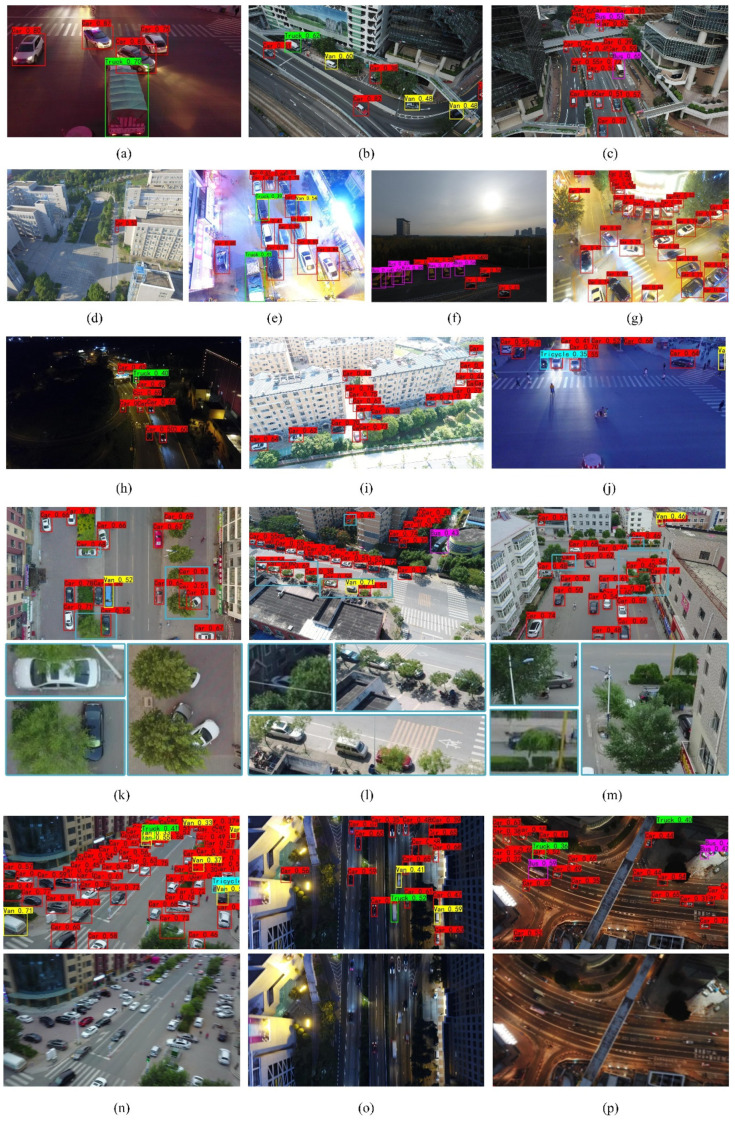
Visualization results of DCNet in the VisDrone dataset. (**a**–**d**) Detection results of DCNet in the case of multiscale changes; (**e**–**j**) are the detection results in the case of illumination variation; (**k**–**m**) are the detection results in the case of occlusion; (**n**–**p**) are the detection results in the blurred case.

In order to explain the performance of the proposed algorithm, DCNet was used on the constructed test dataset. We randomly selected 16 images from the test set (see Figure 6). DCNet featured good performance for scale diversity, blurring, occlusion and illumination. In Figure 6a–d, it can be seen that vehicles of different sizes were detected well. Figure 6c–j show that the detection results under different illumination variations were robust in both dark and bright light environments. The model proposed can still accurately detect the vehicle when the vehicle is occluded by trees and the surrounding environment as shown in Figure 6k–m. Figure 6n–p are the vehicle detection results in a blurred case, which shows that the model also has good performance in blurred inputs.

**Figure 7:**
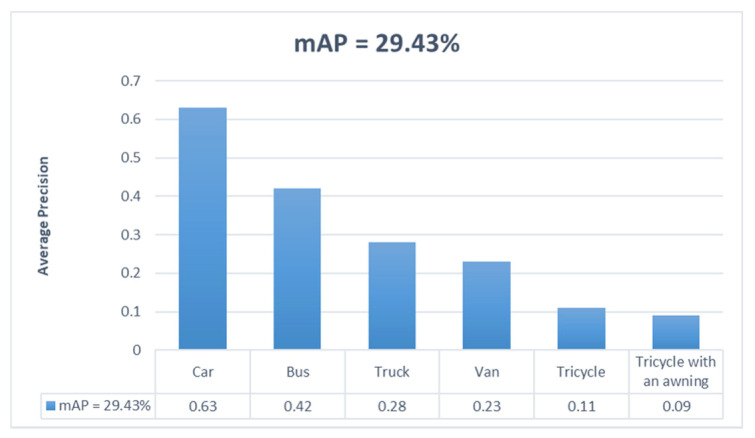
Average precision (AP) values for various categories in the VisDrone dataset and mAP values for DCNet.

The mAP values and the AP values for each category of DCNet in the constructed dataset were computed as shown in Figure 7. The AP values explain that the detection accuracy of the category “car” was the best, with AP values over 0.6.

Although the model has poor detection results for the categories “tricycle” and “tricycle with an awning”, these two categories are the object classes with the smallest number of samples in the dataset; tricycles and tricycles with an awning are smaller than cars and buses in real life; their detection can be improved by adding samples to the dataset.

### 4.3. Comparisons with State-of-the-Art Detectors

We compared the experimental results of the proposed method with state-of-the-art detectors in Table 2. Compared to the baseline detector, our method shows a significant improvement.

As shown in Table 2, the mAP value of Faster R-CNN is the lowest, while the AP value for tricycles is only 1.16%. The reason for this phenomenon is due to the poor detection ability of Faster R-CNN for small target objects. Despite the fact that the SSD method can achieve multiscale detection, the SSD feature mapping is not sufficiently large and has poor performance on the dataset. Because the percentage of the smallest target in the dataset is $2\times 10^{-6},\;$the total recognition pixel size for SSD is 30 pixels. YOLO v3 uses multiple-scale fusion for prediction, which improves the accuracy of detection. However, compared with the DCNet detector, the general detection performance of CenterNet and YOLOv3 in the constructed dataset is lower, which is determined by the complicated and blurred characteristics of UAV images. The experimental results demonstrate that the performance of the DCNet detector is better than that of the others.

**Figure 8:**
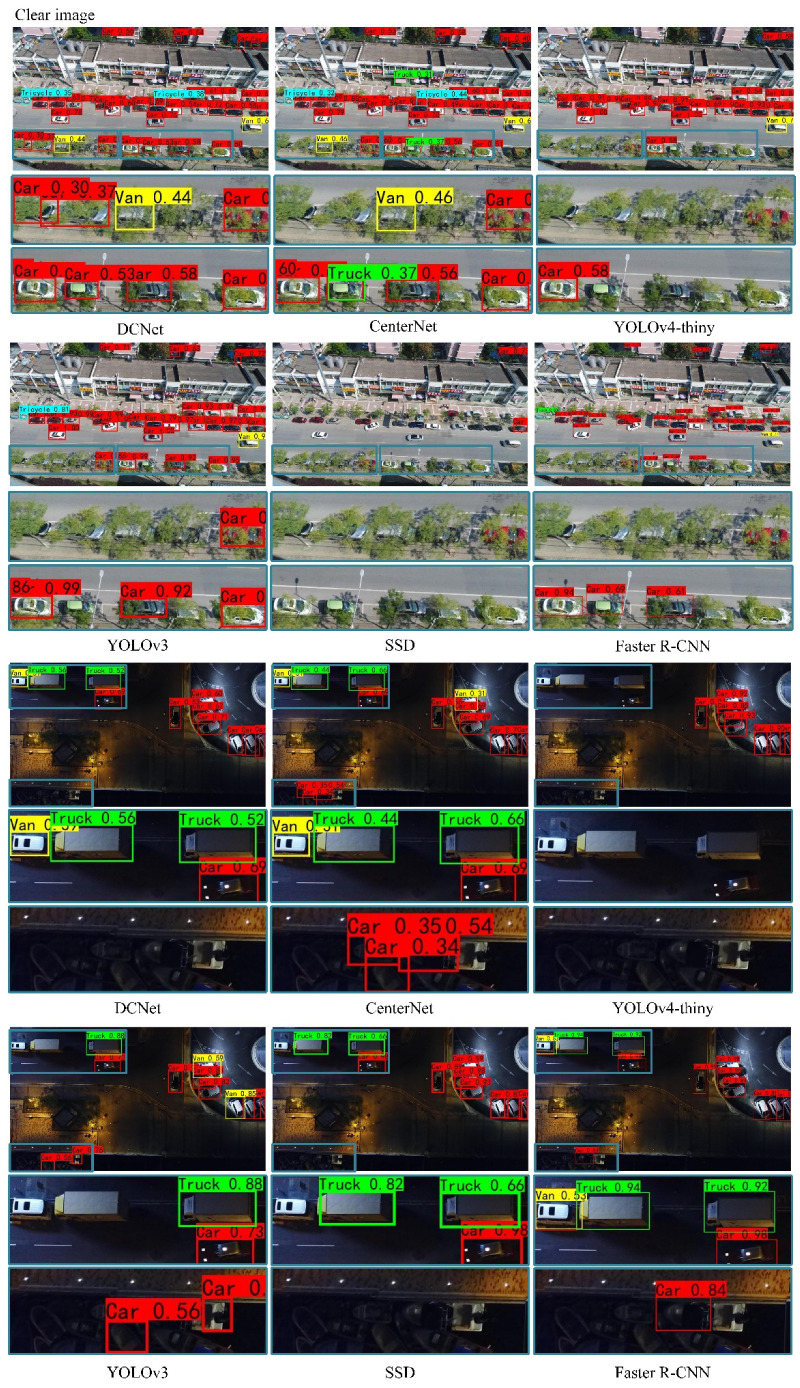

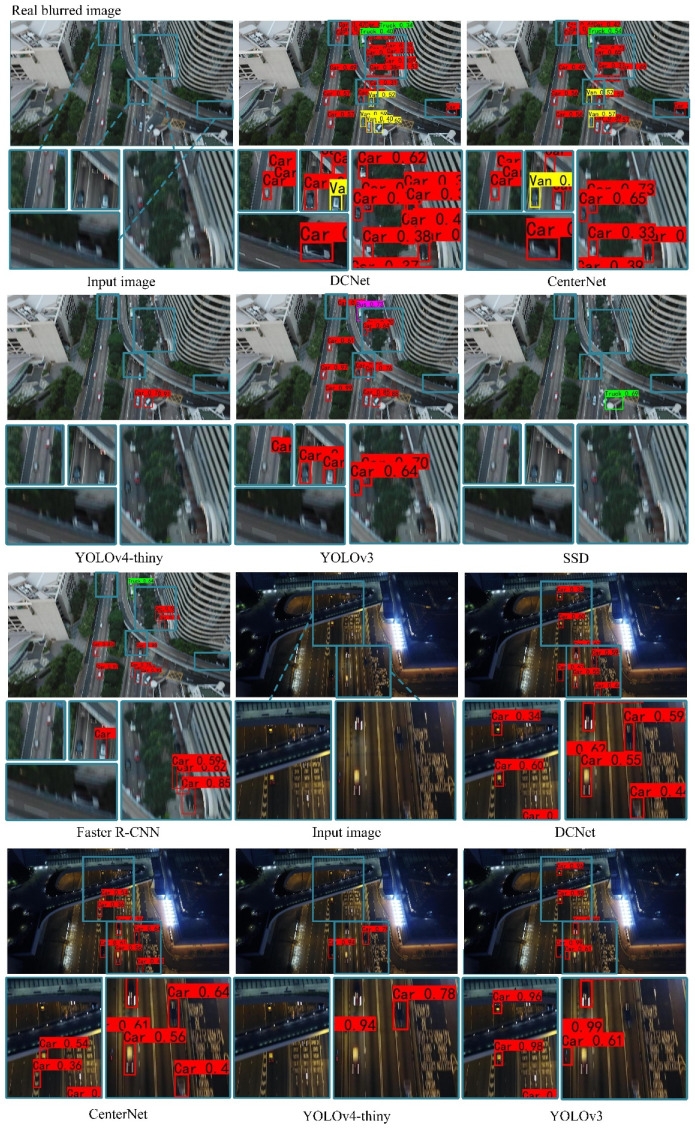

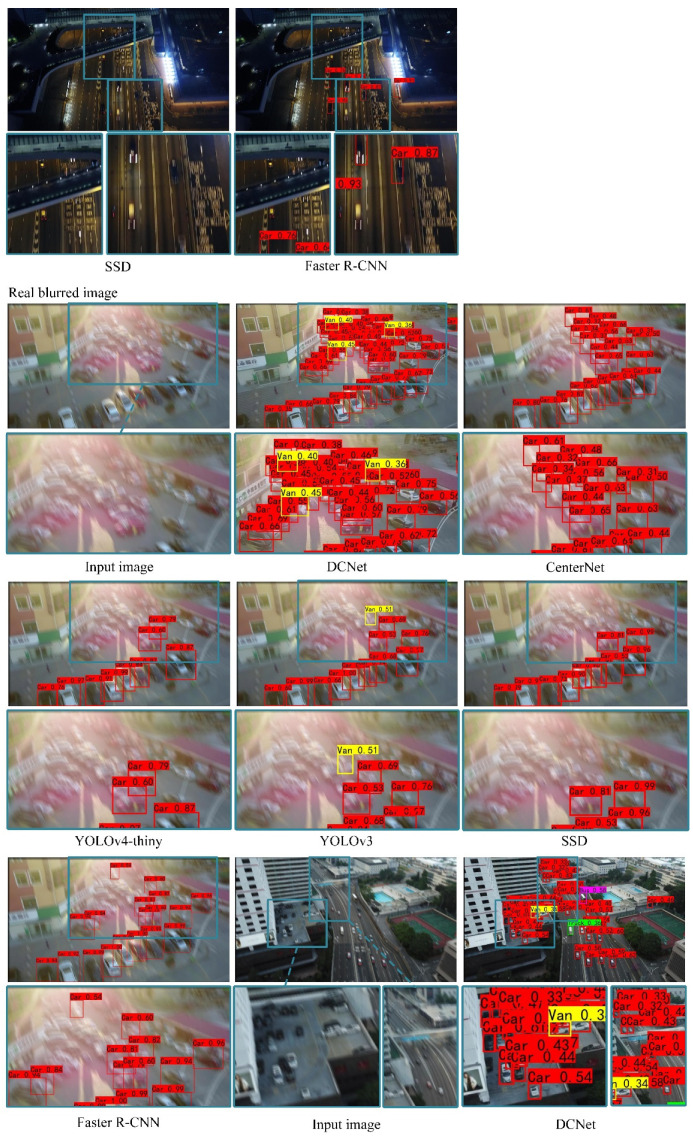

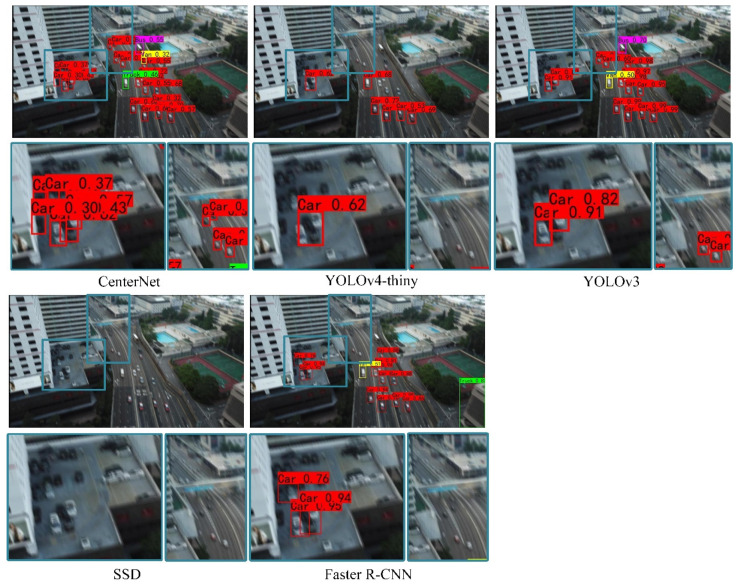
Examples of vehicle detection results using different detection models on three sets of aerial images.

Figure 8 is the visual representation results of object detection for clear images, real blurred images and simulated blurred images. In the case of clear images, the DCNet algorithm can improve the accuracy of vehicle detection (vehicles blocked by trees) and reduce the false detection rate of vehicles (ships falsely detected as vehicles). In the case of real blurred images, the DCNet algorithm can reduce the interference from blurred information, recover the detailed information of the vehicle and ensure a clearer vehicle outline and taillights, which helps to accurately detect the vehicle in a complex road environment. In the case of simulated blurred images, the DCNet algorithm significantly outperforms state-of-the-art detectors, especially in dense areas. DCNet features the best performance in all the three sets of images.

### 4.4. Ablation Experiments

To analyze the importance of the Drone-GAN component, an ablation study was performed in Table 3.

The Drone-GAN module improves mAP by 0.80% (from 28.63% to 29.43%), which means that the module greatly improves the detection performance of motion-blurred targets. Aiming at the problem of target detection that inevitably produces blurred images in the UAV scene, a high-precision model was successfully generated.

The comparative analysis of multiple sets of experiments on the VisDrone dataset demonstrates that the DCNet method achieves good performance in blurred and clear targets detection.

Although the proposed model has obtained relatively good results, it remains a challenge to accurately detect objects in the VisDrone datasets with smaller targets and in more complex scenes. In future work, more accurate detection of small objects with complex scenes will be the key goal.

## 5. Discussion

The experimental results show that DCNet is significantly better than other methods. We believe that the improvement of detection accuracy is mainly brought by the proposed Drone-GAN. Drone-GAN can enhance vehicle features in blurred images, so that vehicles in blurred images can be detected more easily and accurately, which has high robustness towards small target and target occlusion. Compared with our method, Wu et al. [52] proposed a video object detection algorithm based on target blurring degree evaluation, which only considers the blurring degree of the target, but a clear target video frame contributes more to the result than a blurred video frame, thus improving the detection performance. However, this algorithm has poor detection performance when the target is highly blurred, small and occluded. Our method fundamentally solves the problem of blurred images. Hence, our method can achieve superior detection performance, as can be seen from Table 3, and the Drone-GAN module has made a great contribution to the improvement of detection accuracy.

## 6. Conclusions

In this article, due to motion blurring generated by high-speed motion of UAVs or targets, an adaptive deblurring vehicle detection method for high-speed moving drones called DCNet is proposed, which aims to solve the problem of low vehicle detection rate. The CE module based on improved information entropy is used to determine whether the image is blurred. A dataset with blurred aerial images was constructed. The Drone-GAN module is designed to enhance vehicle features in blurred images. The experimental results show that the proposed algorithm can improve the detection accuracy and reduce the false/miss detection rate effectively, enhancing the features of blurred vehicles. The proposed DCNet is robust towards the light variation, occlusion and blurring and can achieve better results in clear and blurred conditions compared with other algorithms. A UAV-captured video sequence processed by the proposed algorithms can be more resistant to jitter. In addition, the proposed module Drone-GAN can be introduced to the image processing pipeline of the UAV’s image sensor to get high-quality motion video. In the future, we plan to detect other specific types of objects such as pedestrians and lane lines, and further study the related domain knowledge to continuously improve target detection in blurred images.

## Figures and Tables

**Table 1:** Example of image scores in the clarity evaluation module.

Clear Image	Entropy Value	Blurred Image	Entropy Value
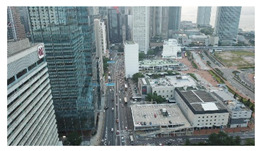	7.15	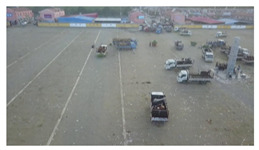	4.85
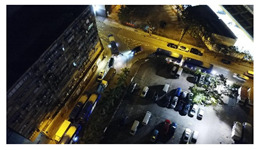	6.19	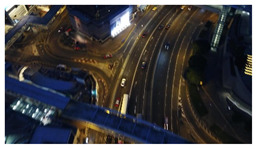	5.31

**Table 2:** The comparison of the mAP values for the use of different methods on the constructed dataset, where the results shown in bold represent the best performance.

Methods	Backbone	Car	Bus	Van	Truck	Tricycle	Tricycle with an Awning	mAP (%)
Faster R-CNN [28]	ResNet-50	25.68	25.80	8.87	14.69	1.16	1.83	13.00
SSD [30]	VGG-16	26.05	30.44	8.40	17.09	2.55	2.97	14.58
YOLOv3 [31]	Darknet53	60.68	41.87	20.37	26.22	**12.98**	6.20	28.05
YOLOv4-thiny [32]	CSPDarknet-thiny	24.67	29.62	5.71	13.73	3.07	1.34	13.02
CenterNet [47]	Hourglass	61.61	40.45	23.01	26.90	11.38	8.42	28.63
DCNet	Hourglass	**62.70**	**42.19**	**23.42**	**28.39**	11.18	**8.72**	**29.43**

**Table 3:** Ablation studies of the VisDrone dataset, where the results shown in bold represent the best performance.

Methods	mAP	F1 Score
Centernet	28.63	28.83
Drone-GAN + CenterNet	**29.43**	**29.50**

## Data Availability

In this work, we exploited the public VisDrone dataset [51] to generate a simulated fuzzy dataset. They can be found from the link: https://github.com/141525/image, accessed on 12 October 2021.

## Abbreviations

The abbreviations in this article are as follows:

SOTA	State of the art
UAV	Unmanned aerial vehicles
GAN	Generative adversarial networks
FOV	Field of view
ICN	Image cascade network
CNN	Convolutional neural network
RPN	Region proposal network
CE	Clarity evaluation
FPN	Feature pyramid network
FA	Feature augmentation
NMS	Non-maximum suppression
YOLO	You only look once
SSD	Single-shot MultiBox detector
VGG	Visual geometry group
GT	Ground truth
ResNet	Residual network
AP	Average precision
mAP	Mean average precision
TP	True-positive
FP	False-positive
FN	False-negative
TN	True-negative

## References

[1] Hua L., Zhang J., Li D., Xi X. (2021). Fault-Tolerant Active Disturbance Rejection Control of Plant Protection of Unmanned Aerial Vehicles Based on a Spatio-Temporal RBF Neural Network. Appl. Sci..

[2] Rajan J., Shriwastav S., Kashyap A., Ratnoo A., Ghose D. (2021). Disaster management using unmanned aerial vehicles. Unmanned Aerial Systems.

[3] Shrestha R., Bajracharya R., Kim S. (2021). 6G Enabled Unmanned Aerial Vehicle Traffic Management: A Perspective. IEEE Access.

[4] Lee G., Hwang J., Cho S. (2021). A Novel Index to Detect Vegetation in Urban Areas Using UAV-Based Multispectral Images. Appl. Sci..

[5] Ko Y.D., Song B.D. (2021). Application of UAVs for tourism security and safety. Asia Pac. J. Mark. Logist..

[6] Delavarpour N., Koparan C., Nowatzki J., Bajwa S., Sun X. (2021). A Technical Study on UAV Characteristics for Precision Agriculture Applications and Associated Practical Challenges. Remote Sens..

[7] Barbosa B., Ferraz G.A.E.S., dos Santos L.M., Santana L., Marin D.B., Rossi G., Conti L. (2021). Application of RGB Images Obtained by UAV in Coffee Farming. Remote Sens..

[8] Yahia C.N., Scott S.E., Boyles S.D., Claudel C.G. (2021). Unmanned aerial vehicle path planning for trafc estimation and detection of non-recurrent congestion. Transp. Lett..

[9] Anagnostopoulos A., Kehagia F. (2021). Utilizing UAVs Technology on Microscopic Traffic Naturalistic Data Acquirement. Infrastructures.

[10] Lin T.Y., Dollár P., Girshick R., He K., Hariharan B., Belongie S. Feature pyramid networks for object detection. Proceedings of the IEEE Conference on Computer Vision and Pattern Recognition.

[11] Azimi S.M., Vig E., Bahmanyar R., Körner M., Reinartz P. (2019). Towards Multi-Class Object Detection in Unconstrained Remote Sensing Imagery.

[12] LaLonde R., Zhang D., Shah M. ClusterNet: Detecting Small Objects in Large Scenes by Exploiting Spatio-Temporal Information. Proceedings of the 2018 IEEE/CVF Conference on Computer Vision and Pattern Recognition.

[13] Yang F., Fan H., Chu P., Blasch E., Ling H. Clustered Object Detection in Aerial Images. Proceedings of the 2019 IEEE/CVF International Conference on Computer Vision (ICCV).

[14] Regester A., Paruchuri V. Using Computer Vision Techniques for Parking Space Detection in Aerial Imagery. Proceedings of the Advances in Intelligent Systems and Computing.

[15] Guido G., Gallelli V., Rogano D., Vitale A. (2016). Evaluating the accuracy of vehicle tracking data obtained from Unmanned Aerial Vehicles. Int. J. Transp. Sci. Technol..

[16] Sakai K., Seo T., Fuse T. Traffic density estimation method from small satellite imagery: Towards frequent remote sensing of car traffic. Proceedings of the 2019 IEEE Intelligent Transportation Systems Conference (ITSC).

[17] Moranduzzo T., Melgani F. A SIFT-SVM method for detecting cars in UAV images. Proceedings of the 2012 IEEE International Geoscience and Remote Sensing Symposium.

[18] Kembhavi A., Harwood D., Davis L.S. (2010). Vehicle Detection Using Partial Least Squares. IEEE Trans. Pattern Anal. Mach. Intell..

[19] Cao X., Wu C., Yan P., Li X. Linear SVM classification using boosting HOG features for vehicle detection in low-altitude airborne videos. Proceedings of the 2011 18th IEEE International Conference on Image Processing.

[20] Moranduzzo T., Melgani F. (2014). Detecting Cars in UAV Images with a Catalog-Based Approach. IEEE Trans. Geosci. Remote Sens..

[21] Zhou H., Wei L., Lim C.P., Creighton D., Nahavandi S. (2018). Robust Vehicle Detection in Aerial Images Using Bag-of-Words and Orientation Aware Scanning. IEEE Trans. Geosci. Remote Sens..

[22] Moranduzzo T., Melgani F. Comparison of different feature detectors and descriptors for car classification in UAV images. Proceedings of the 2013 IEEE International Geoscience and Remote Sensing Symposium—IGARSS.

[23] Shao W., Yang W., Liu G., Liu J. Car detection from high-resolution aerial imagery using multiple features. Proceedings of the Geoscience and Remote Sensing Symposium (IGARSS), 2012 IEEE International.

[24] Liang P., Teodoro G., Ling H., Blasch E., Chen G., Bai L. Multiple kernel learning for vehicle detection in wide area motion imagery. Proceedings of the 2012 15th International Conference on Information Fusion.

[25] Cheng H.-Y., Weng C.-C., Chen Y.-Y. (2011). Vehicle Detection in Aerial Surveillance Using Dynamic Bayesian Networks. IEEE Trans. Image Process..

[26] Girshick R., Donahue J., Darrell T., Malik J. Rich feature hierarchies for accurate object detection and semantic segmentation. Proceedings of the IEEE Conference on Computer Vision and Pattern Recognition.

[27] Girshick R.J.C.S. Fast R-CNN. Proceedings of the IEEE International Conference on Computer Vision.

[28] Ren S., He K., Girshick R., Sun J. (2017). Faster R-CNN: Towards real-time object detection with region proposal networks. IEEE Trans. Pattern Anal. Mach. Intell..

[29] Redmon J., Divvala S., Girshick R., Farhadi A. You Only Look Once: Unified, Real-Time Object Detection. Proceedings of the IEEE Conference on Computer Vision and Pattern Recognition (CVPR).

[30] Liu W., Anguelov D., Erhan D., Szegedy C., Reed S., Fu C.Y., Berg A.C. (2016). SSD: Single Shot MultiBox Detector. European Conference on Computer Vision.

[31] Redmon J., Farhadi A. (2018). YOLOv3: An Incremental Improvement. arXiv.

[32] Wang C.Y., Bochkovskiy A., Liao H.Y.M. Scaled-YOLOv4: Scaling Cross Stage Partial Network. Proceedings of the IEEE/CVF Conference on Computer Vision and Pattern Recognition Virtual.

[33] Ji H., Gao Z., Mei T., Ramesh B. (2020). Vehicle Detection in Remote Sensing Images Leveraging on Simultaneous Super-Resolution. IEEE Geosci. Remote Sens. Lett..

[34] Rabbi J., Ray N., Schubert M., Chowdhury S., Chao D. (2020). Small-Object Detection in Remote Sensing Images with End-to-End Edge-Enhanced GAN and Object Detector Network. Remote Sens..

[35] Singh B., Davis L.S. An Analysis of Scale Invariance in Object Detection—SNIP. Proceedings of the 2018 IEEE/CVF Conference on Computer Vision and Pattern Recognition.

[36] Mostofa M., Ferdous S.N., Riggan B.S., Nasrabadi N.M. (2020). Joint-SRVDNet: Joint Super Resolution and Vehicle Detection Network. IEEE Access.

[37] Mandal M., Shah M., Meena P., Devi S., Vipparthi S.K. (2019). AVDNet: A Small-Sized Vehicle Detection Network for Aerial Visual Data. IEEE Geosci. Remote Sens. Lett..

[38] Bai J.J., Hong C.Y. (2009). Edge Detect Based on Sobel. Comput. Knowl. Technol..

[39] Qi L., Huajun F., Zhihai X., Meijuan B., Su S., Ruichun D. (2002). Digital image sharpness evaluation function. Acta Photonica Sin..

[40] Kupyn O., Budzan V., Mykhailych M., Mishkin D., Matas J. DeblurGAN: Blind Motion Deblurring Using Conditional Adversarial Networks. Proceedings of the 2018 IEEE/CVF Conference on Computer Vision and Pattern Recognition.

[41] Boracchi G., Foi A. (2012). Modeling the Performance of Image Restoration From Motion Blur. IEEE Trans. Image Process..

[42] Goodfellow I.J., Pouget-Abadie J., Mirza M., Xu B., Warde-Farley D., Ozair S., Courville A., Bengio Y. Generative Adversarial Networks. In Proceedings the 27th International Conference on Neural Information Processing Systems, Palais des Congrès de Montréal.

[43] Szegedy C., Ioffe S., Vanhoucke V., Alemi A.A. Inception-v4, Inception-ResNet and the Impact of Residual Connections on Learning. Proceedings of the Thirty-First AAAI Conference on Artificial Intelligence.

[44] Jolicoeur-Martineau A. (2018). The relativistic discriminator: A key element missing from standard GAN. arXiv.

[45] Mao X., Li Q., Xie H., Lau R.Y.K., Wang Z., Smolley S.P. (2019). On the Effectiveness of Least Squares Generative Adversarial Networks. IEEE Trans. Pattern Anal. Mach. Intell..

[46] Isola P., Zhu J.-Y., Zhou T., Efros A.A. Image-to-Image Translation with Conditional Adversarial Networks. Proceedings of the 2017 IEEE Conference on Computer Vision and Pattern Recognition (CVPR).

[47] Zhou X., Wang D., Krähenbühl P. (2019). Objects as points. arXiv.

[48] Neubeck A., Van Gool L. Efficient non-maximum suppression. In Proceeding of the 18th International Conference on Pattern Recognition (ICPR’06).

[49] Newell A., Yang K., Deng J. (2016). Stacked Hourglass Networks for Human Pose Estimation. European Conference on Computer Vision.

[50] Lin T.Y., Goyal P., Girshick R., He K., Dollár P. Focal Loss for Dense Object Detection. Proceedings of the IEEE International Conference on Computer Vision.

[51] Zhu P., Du D., Wen L., Bian X., Ling H., Hu Q., Peng T., Zheng J., Wang X., Zhang Y. VisDrone-VID2019: The Vision Meets Drone Object Detection in Video Challenge Results. Proceedings of the IEEE/CVF International Conference on Computer Vision Workshops.

[52] Wu Y., Zhang H., Li Y., Yang Y., Yuan D. (2020). Video Object Detection Guided by Object Blur Evaluation. IEEE Access.

